# Organ-specific depth of response to CD19-directed CAR T-cell therapy in B-cell lymphomas

**DOI:** 10.1007/s00262-026-04605-7

**Published:** 2026-10-25

**Authors:** Matthias Kassube, Philipp Achhammer, Philipp Linden, Veit L. Bücklein, Tobias Tix, Linus Kruk, Adrien Holzgreve, Rudolf A. Werner, Jens Ricke, Michael von Bergwelt-Baildon, Kai Rejeski, Marion Subklewe, Wolfgang G. Kunz, Michael Winkelmann

**Affiliations:** 1 Department of Radiology, University Hospital, LMU Munich, Marchioninistr. 15, 81377 Munich, Germany; 2 Laboratory for Translational Cancer Immunology, Gene Center of the LMU Munich, Munich, Germany; 3 Partner Site Munich, German Cancer Consortium (DKTK) and Bavarian Center for Cancer Research (BZKF), Munich, Germany; 4 Department of Medicine III , University Hospital, LMU Munich, Munich, Germany; 5 Department of Nuclear Medicine, University Hospital, LMU Munich, Munich, Germany; 6 Comprehensive Cancer Center München-LMU (CCCMLMU), LMU Munich, Munich, Germany; 7 Laboratory of Precision Immunotherapy, Department of Medicine III, LMU University Hospital, LMU Munich, Munich, Germany

**Keywords:** CAR T-cell therapy, Lymphoma, Organ-specific, Depth of response

## Abstract

**Background:**

CD19-directed chimeric antigen receptor T cell therapy (CART) has transformed treatment of relapsed or refractory (r/r) large B cell lymphoma (LBCL); however, organ-specific response patterns and depth of response (DoR) remain insufficiently characterized. This study evaluated organ-specific imaging response and the impact of spatial disease patterns on progression-free survival (PFS) and overall survival (OS).

**Materials and methods:**

In this retrospective single-center study, 109 consecutive patients with r/r LBCL treated with CD19-directed CART between January 2019 and May 2025 were included. Baseline and follow-up CT or PET/CT was analyzed using structured lesion tracking. Up to six target lesions per patient were assessed according to Lugano criteria, and the sum of the products of diameters (SPD) was calculated. DoR was defined as percentage change in SPD from baseline. Associations with PFS and OS were evaluated using Kaplan–Meier analysis and Cox regression.

**Results:**

Among 109 patients (median age, 65 years [IQR, 57–71]), 65 (59.6%) had extranodal disease at baseline. Baseline tumor burden was higher in patients with extranodal than nodal disease (median SPD, 3573 mm^2^ [IQR, 1314–9708] versus 1998 mm^2^ [IQR, 1347–2921]). DoR was comparable between nodal and extranodal lesions at both time points (− 62.1% vs. − 65.6% and − 77.9% vs. − 78.1%); this comparability at the second follow-up may partly reflect differential attrition, as extranodal patients were more likely to be lost to follow-up before this timepoint. Despite similar tumor reduction, median PFS was 306.5 days in nodal versus 90 days in extranodal disease. Baseline tumor burden (SPD) was independently associated with both PFS and OS (HR per doubling of SPD 1.17 and 1.21, respectively). Pleural, hepatobiliary, and adrenal involvement showed the strongest associations with adverse outcomes in this exploratory, hypothesis-generating organ-level analysis; pleural involvement remained independently associated with PFS and OS, though organ-specific subgroups were small (*n* = 7–19) and estimates should be interpreted with caution.

**Conclusion:**

Tumor reduction occurs in nodal and extranodal disease after CART, but outcomes depend on anatomical disease distribution. Pleural and hepatobiliary involvement emerged as candidate high-risk imaging phenotypes in this exploratory analysis, warranting confirmation in larger, multicenter cohorts. Organ-specific assessment may provide information beyond global tumor burden.

**Supplementary Information:**

The online version contains supplementary material available at https://doi.org/10.1007/s00262-026-04605-7.

## Introduction

Chimeric antigen receptor T cell therapy (CART) directed against CD19 has revolutionized the treatment of relapsed or refractory (r/r) B cell non-Hodgkin lymphomas [1–4]. In aggressive lymphomas like diffuse large B cell lymphoma (DLBCL), CART infusions can induce high initial remission rates even in chemorefractory cases, transforming the outlook for many patients [5]. Despite these advances, between 30 and 60% of lymphoma patients ultimately experience disease relapse or progression after CART [5]. This highlights the need to understand tumor- and host-specific factors that limit long-term efficacy. One such factor of growing interest is the anatomical distribution of lymphoma, particularly extranodal disease (END) involvement at the time of CART infusion [5].

Extranodal disease is a common feature of advanced B cell lymphomas and has significant relevance for clinical management and outcomes [6]. In first-line DLBCL management, the presence of END has long been recognized as an adverse prognostic feature, especially when critical organs such as the central nervous system (CNS), lungs, liver, or pancreas are involved [7, 8]. Patients with extensive END often have aggressive tumor biology; historically, END DLBCL was associated with inferior survival outcomes compared to purely nodal disease [8–10]. Also in the setting of CART, involvement of two or more extranodal organs represents a risk factor with significant impact on prognosis [11].

A recent retrospective analysis of adults with large B cell lymphoma (LBCL) treated with commercial CD19-directed CART demonstrated that END at baseline was highly prevalent and had a significant impact on treatment outcomes. While patients with isolated END showed similar outcomes to those with nodal disease alone, individuals with concomitant END and nodal involvement exhibited markedly lower complete response rates and shorter progression-free survival (PFS). Importantly, response and relapse rates varied substantially by anatomical site of END involvement, with particularly low local response rates observed in disease involving the lungs/pleura/pericardium and gastrointestinal/peritoneum [5].

In addition to END, depth of response (DoR), defined as the magnitude of tumor burden reduction after therapy, is an established prognostic indicator across lymphoid malignancies, with deeper responses generally translating into improved long-term outcomes including PFS and overall survival (OS). In non-Hodgkin lymphomas treated with conventional chemo-immunotherapy, patients achieving complete remission consistently show superior PFS compared with those achieving only partial responses [12, 13]. Emerging evidence in the context of CD19-directed CART indicates a similar pattern, where deep initial responses, especially complete metabolic remissions, are linked with more favorable long-term outcomes [14]. Moreover, imaging-based metrics of response depth using structured criteria (e.g., Lugano or RECIL) can stratify patients by subsequent PFS and OS outcomes [15].

Despite the recognized prognostic relevance of END distribution and DoR as independent factors, their combined assessment, particularly organ-specific DoR following CART, has not yet been systematically investigated. Therefore, the aim of the present study was to comprehensively characterize organ-specific DoR in patients undergoing CART and to evaluate its association with clinical outcomes including PFS and OS.

## Methods

### Study design and population

All medical records and imaging studies underwent review with approval from the LMU Munich Institutional Review Board (LMU Ethics Committee, project number 19–817). All individual patients gave informed consent. The study population comprised all consecutive patients treated with standard-of-care CD19-specific CART products at the Comprehensive Cancer Center Munich–LMU (CCCMLMU) between January 2019 and May 2025 and was analyzed retrospectively. Extranodal disease was defined as involvement of any site outside the lymph nodes, Waldeyer’s ring, and spleen. (Splenic involvement was classified as nodal disease.)

Inclusion criteria included patients with r/r LBCL, available CT or PET/CT imaging at baseline (≤ 2 weeks before CART), and any measurable disease according to Lugano criteria [16]. Exclusion criteria included no measurable disease on imaging and any non-diagnostic or incomplete imaging studies.

Histologic diagnoses were evaluated by expert pathologists. Patients underwent lymphodepletion with fludarabine and cyclophosphamide according to manufacturers’ instructions. Bridging therapy was defined as systemic therapy or local radiation between time of indication and lymphodepletion. Systematic data on peri-CART radiotherapy (bridging or consolidative) were not captured in our structured registry and could therefore not be incorporated into this analysis (see Limitations). The International Prognostic Index (IPI) was calculated using age, Eastern Cooperative Oncology Group (ECOG) performance status, Ann Arbor stage, serum lactate dehydrogenase (LDH), and extranodal involvement.

### Imaging and response assessment

All patients underwent baseline cross-sectional imaging with CT or 18F-fluorodeoxyglucose PET/CT prior to CART infusion. Follow-up imaging 1 (FU1) was performed at approximately 1 month (± 2 weeks) and second follow-up imaging (FU2) at 3 months (± 4 weeks) post-infusion. Lesion assessment was performed using MINT Lesion™ (Mint Medical GmbH, Heidelberg, Germany). In line with Lugano 2014 response criteria, up to six target lesions were selected per patient, measured bidimensionally, and the SPD was calculated. SPD was calculated from the CT component of either CT or PET/CT using identical measurement methodology, eliminating measurement-level bias between modalities; differential lesion detection sensitivity between CT and PET/CT (e.g., for FDG-avid but structurally inconspicuous lesions) nonetheless represents an inherent limitation of a mixed-modality cohort (see Limitations).

### Longitudinal assessment of extranodal involvement and depth of response

Extranodal involvement was systematically assessed at baseline and during follow-up. For each involved organ, the SPD was calculated according to the Lugano classification. Longitudinal assessment of extranodal disease burden was performed by tracking organ-specific SPD values at first (FU1) and second (FU2) follow-up examinations. DoR was calculated as the percentage change in SPD relative to baseline.

### Statistical analysis

Statistical analyses were conducted using GraphPad Prism (version 10) and R version 4.5.2. Continuous variables were reported as median with interquartile range (IQR). LDH values were expressed relative to the local upper limit of normal (250 U/L). Changes in SPD and DoR across timepoints were assessed using paired statistical tests (paired *t* test or Wilcoxon signed-rank test, depending on distribution). Survival analyses for PFS and OS were performed using Kaplan–Meier methodology with log-rank comparisons. Cox regression was used for univariable and multivariable analyses. A *p*-value < 0.05 was considered statistically significant. Given that 16 organ sites were evaluated across two endpoints (32 comparisons) without correction for multiple testing, organ-level results are reported descriptively and should be interpreted as exploratory and hypothesis-generating rather than confirmatory. Events-per-variable (EPV) for the multivariable models are reported in the Results.

## Results

### Patient cohort and baseline characteristics

**Fig. 1:**
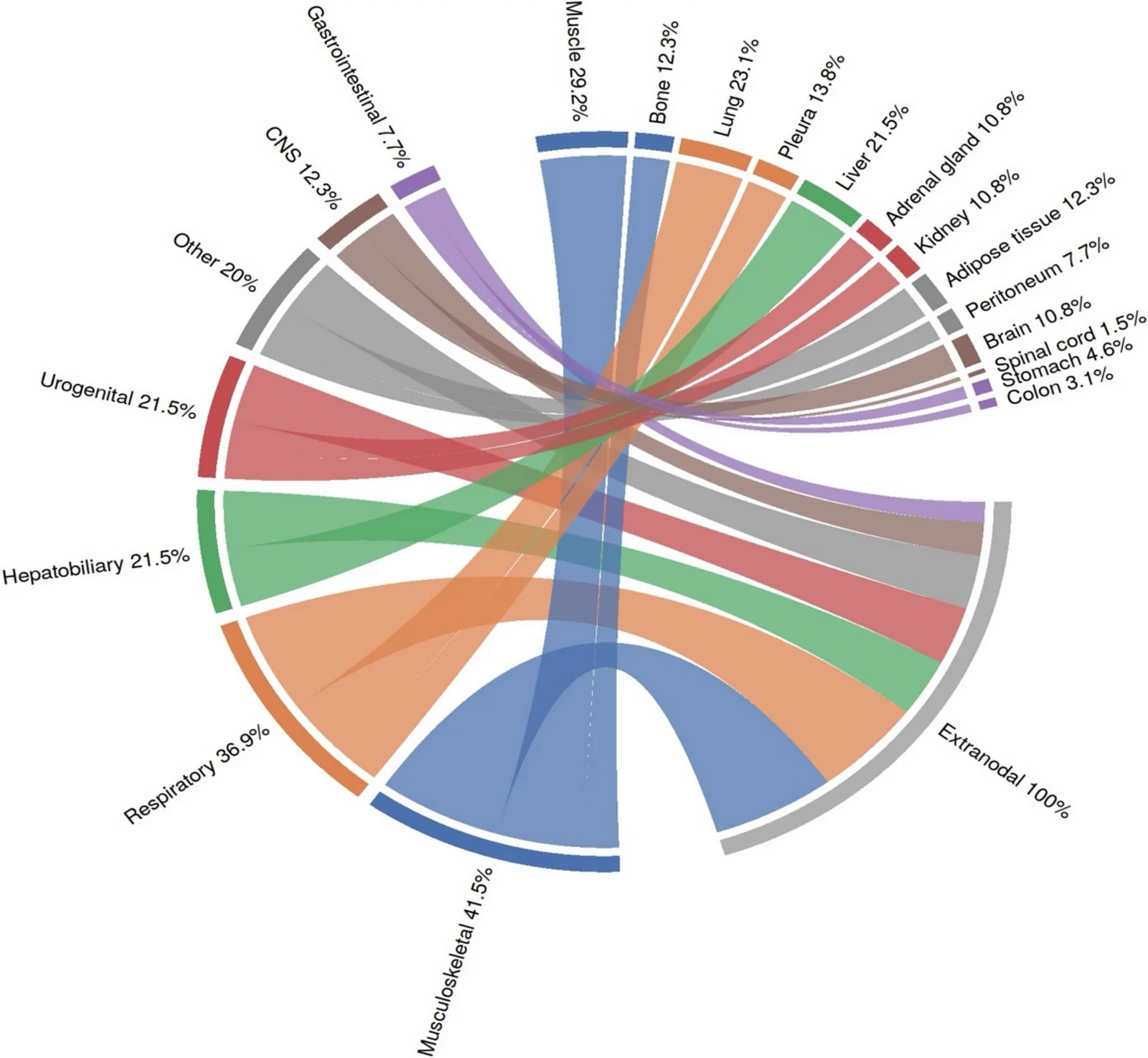
Distribution of extranodal involvement across organ systems and organs. Circular Sankey diagram illustrating the distribution of extranodal disease. The width of each flow represents the relative frequency of involvement

A total of 109 patients with LBCL with available baseline imaging were included. Of these, 102 patients underwent first follow-up imaging at 30 days after CART, and 84 patients had second follow-up imaging at 3 months post-infusion. A detailed study flowchart is provided in Supplementary Figure S1. The cohort comprised 44 patients with purely nodal disease and 65 with extranodal involvement. Among patients with extranodal disease, involvement of a single extranodal organ was most common (27.5%), followed by two (22.0%), three (7.3%), and four organs (1.8%). The distribution of affected organ systems is shown in Fig. 1.

The median age was 65 years (IQR, 57–71) and was comparable between groups. Overall, 54.1% of patients were male. Stage IV disease was observed only in patients with extranodal involvement; however, not all patients with extranodal disease met Stage IV criteria, reflecting cases of localized single-site extranodal disease (Ann Arbor E-lesion) without diffuse dissemination. (Stage IV accounted for 55.4% of the extranodal subgroup; Table 1.) LDH levels were higher in patients with extranodal disease (median, 349 U/L [IQR, 279–396]) compared with nodal disease (235 U/L [IQR, 193–332]). Most patients had favorable performance status (ECOG 0–1, 79.8%). The IPI distribution showed a predominance of intermediate-risk disease (IPI 2–3, 51.8%). Detailed baseline characteristics are summarized in Table 1.

**Table 1:** Patient characteristics

Variable		All	Pure nodal	Extranodal
N		109	44	65
Age		65 (57–71)	65 (56–71)	66 (60–67)
Sex	Male	59 (54.1%)	21 (47.7%)	38 (58.5%)
	Female	50 (45.9%)	23 (52.3%)	27 (41.5%)
CAR-T product	Axi-cel	63 (57.8%)	26 (59.1%)	37 (56.9%)
	Tisa-cel	33 (30.3%)	12 (27.3%)	21 (32.3%)
	Liso-cel	12 (11.0%)	5 (11.4%)	7 (10.8%)
	Zamto-cel*	1 (0.9%)	1 (2.3%)	0
SPD in mm^2^	BL	2921 (2221–3956)	1998 (1347–2921)	3573 (1314–9708)
	FU1	1284 (717–1633)	757.9 (416.3–1359)	1717 (379–4587)
	FU2	661 (366–1134)	442.6 (209.4–1066)	969.1 (207.8–2747)
Ann-Arbor	1	15 (13.8%)	8 (18.2%)	7 (10.8%)
	2	32 (29.4%)	18 (40.9%)	14 (21.5%)
	3	26 (23.9%)	18 (40.9%)	8 (12.3%)
	4	36 (33.0%)	0	36 (55.4%)
LDH		283 (251–340)	235 (193–332)	349 (279–396)
ECOG	0	29 (26.6%)	17 (38.6%)	12 (18.5%)
	1	58 (53.2%)	22 (50.0%)	36 (55.4%)
	2	13 (11.9%)	2 (4.6%)	11 (16.9%)
	3	7 (6.4%)	3 (6.8%)	4 (6.2%)
	4	0	0	0
> 2EN		39 (35.78%)	0	39 (60.0%)
IPI	0	8 (7.3%)	6 (13.6%)	2 (3.1%)
	1	27 (24.8%)	16 (36.4%)	13 (20.0%)
	2	22 (20.2%)	10 (22.7%)	12 (18.5%)
	3	29 (26.6%)	11 (25.0%)	18 (27.7%)
	4	15 (13.8%)	1 (2.3%)	14 (21.5%)
	5	8 (7.3%)	0	8 (12.3%)
Depth of response	BL–FU1	− 56.0%	− 62.1%	− 65.6%
	BL–FU2	− 77.4%	− 77.9%	− 78.1%
PFS days		130 (90–260)	306.5 (153–418)	90 (70–130)
OS days		356 (251–532)	613 (333–860)	247 (169–384)
Response FU1	N	102	42	60
	CR	29 (28.4%)	15 (35.7%)	14 (23.3%)
	PR	36 (35.3%)	17 (40.5%)	19 (31.7%)
	SD	16 (15.7%)	6 (14.3%)	10 (16.7%)
	PD	21 (20.6%)	4 (9.5%)	17 (28.3%)
Response FU2	N	84	37	47
	CR	44 (52.4%)	23 (62.2%)	21 (44.6%)
	PR	10 (11.9%)	5 (13.5%)	5 (10.6%)
	SD	2 (2.3%)	0	2 (4.3%)
	PD	28 (33.3%)	9 (24.3%)	19 (40.4%)

^*^Zamtocabtagene autoleucel, an investigational CD19 CART construct administered within a clinical trial. Product distribution reflects sequential clinical availability over the study period rather than random allocation across groups (see “Discussion” in section)

### Tumor burden, depth of response, and overall response

**Fig. 2:**
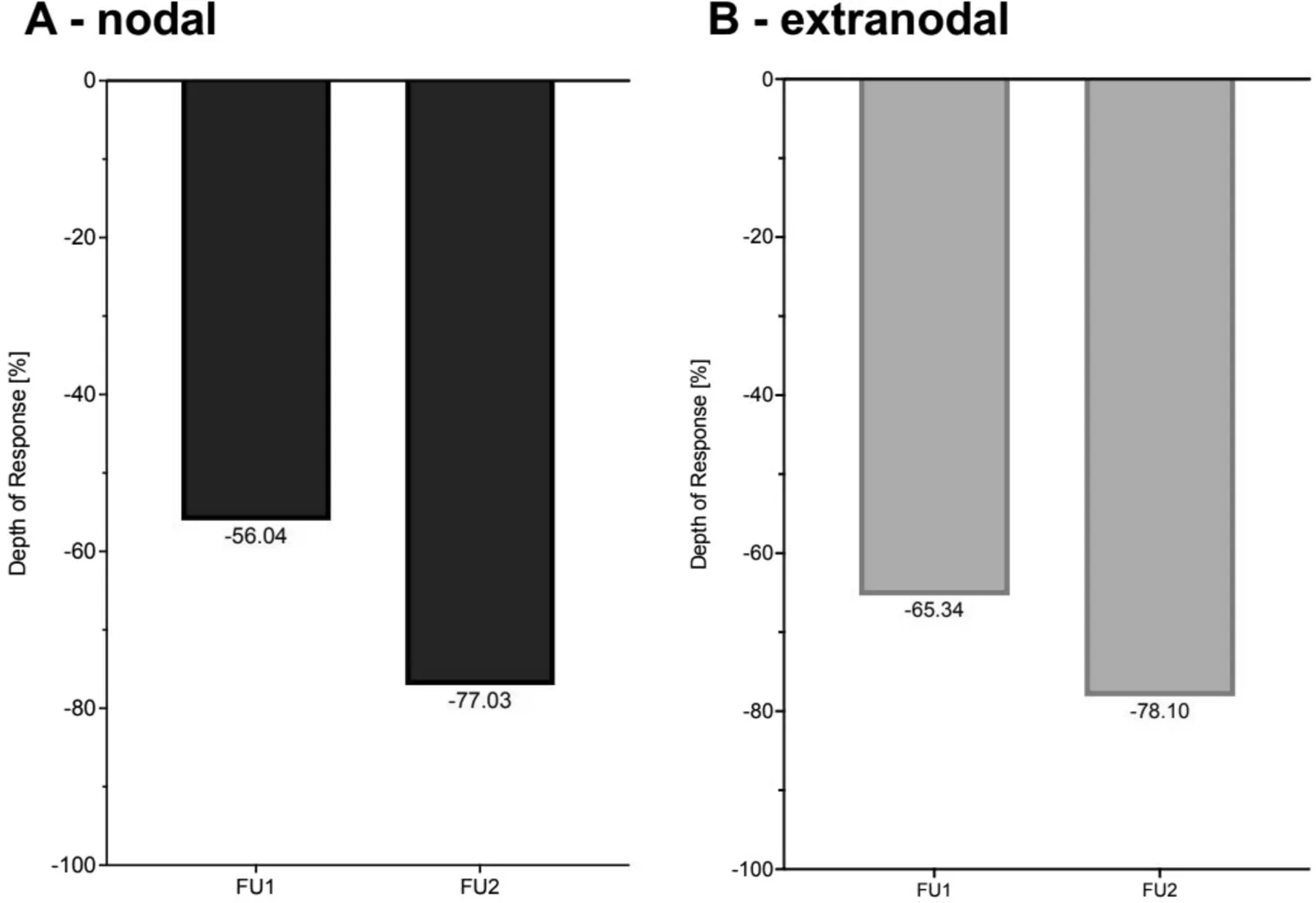
Depth of response (DoR) during follow-up. Bar plots illustrate the median percentage reduction in tumor burden at FU1 and FU2 for nodal (left) and extranodal (right) disease

Baseline tumor burden (SPD) was higher in patients with extranodal disease (median, 3573 mm^2^ [IQR, 1314–9708]) than in those with nodal disease (1998 mm^2^ [IQR, 1347–2921]). Median SPD declined to 1284 mm^2^ (IQR, 717–1633) at FU1 and further to 661 mm^2^ (IQR, 366–1134) at FU2. At the lesion level, nodal and extranodal lesions demonstrated comparable responses. From baseline to FU1, the median DoR was − 62.1% in nodal lesions and − 65.6% in extranodal lesions; from baseline to FU2, it increased to − 77.9% and − 78.1%, respectively (Fig. 2). Extranodal patients were substantially more likely to be excluded from second follow-up (FU2) imaging than nodal patients (18/65 [27.7%] vs. 5/44 [11.4%]); among patients missing FU2 imaging, 91% had died by the data cutoff (median OS 51 vs. 531 days for patients without vs. with FU2 imaging). This differential attrition likely biases the FU2 depth-of-response comparison toward apparent similarity, as the deepest and most durable responders are disproportionately the ones who survive to be re-imaged.

**Fig. 3:**
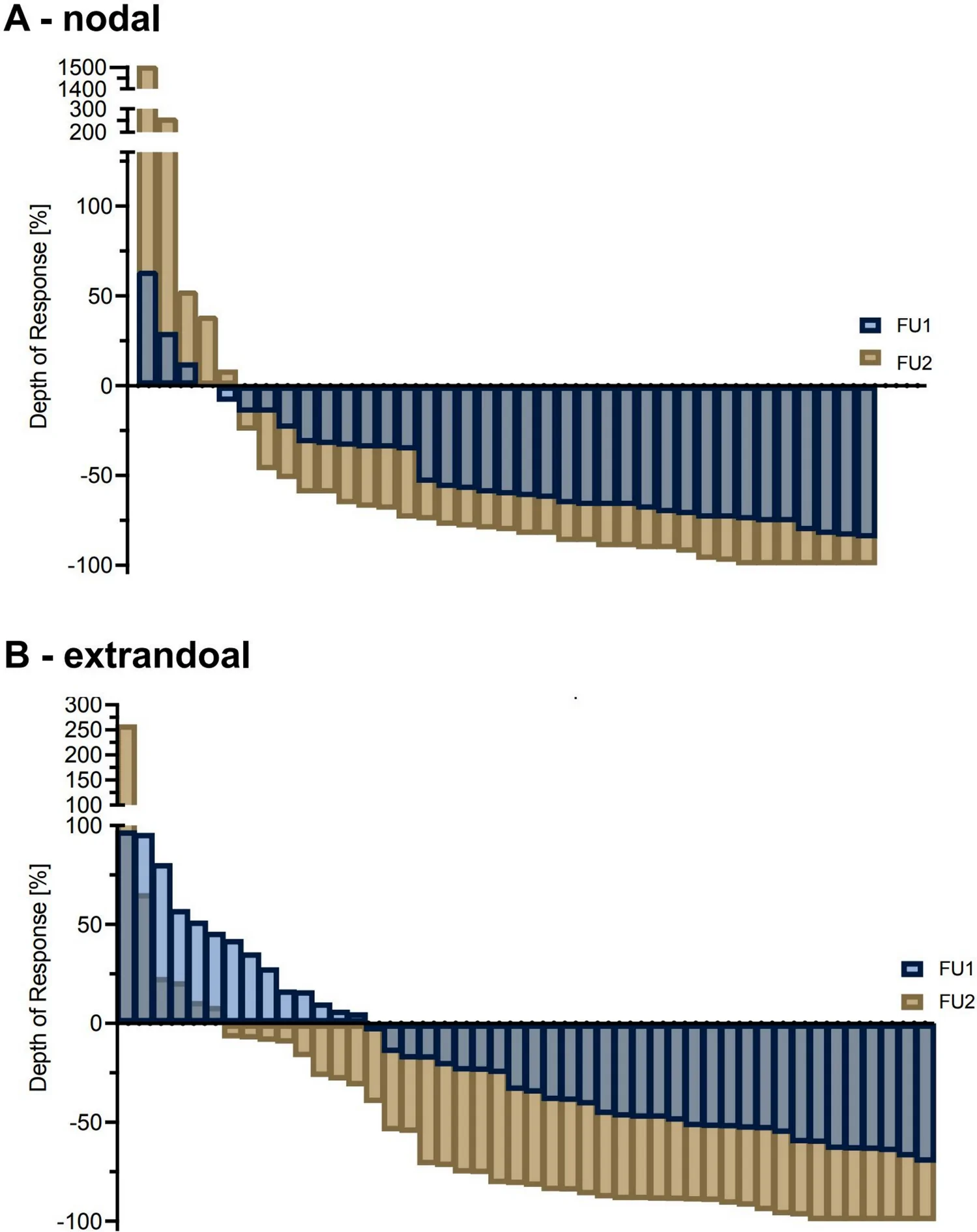
Individual patient DoR in nodal and extranodal disease. Waterfall plots illustrate the percentage change in tumor burden at FU1 and FU2 for nodal (on top) and extranodal (below) disease

At FU1, complete response (CR) occurred in 28.4%, partial response (PR) in 35.3%, stable disease (SD) in 15.7%, and progressive disease (PD) in 20.6%. At FU2, CR increased to 52.4%, PR to 11.9%, SD to 2.3%, and PD to 33.3%. CR rates were higher in nodal disease (62.2%) than extranodal disease (44.6%). Waterfall plots illustrate individual DoR (Fig. 3).

### Organ-specific response

**Fig. 4:**
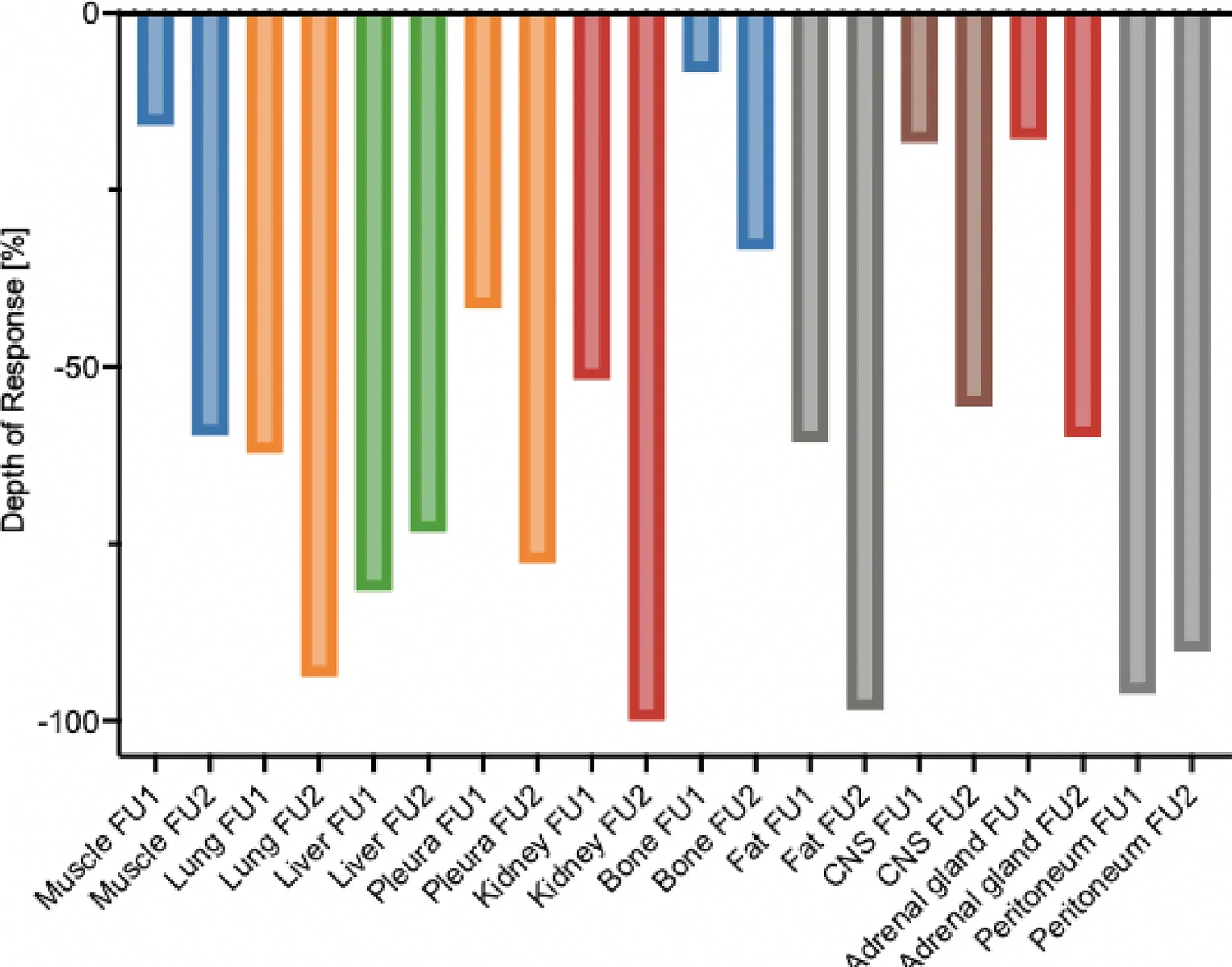
Depth of response by extranodal organ system. Bar plots illustrate the percentage change in tumor burden for different extranodal organ systems at FU1 and FU2

Depth of response was analyzed by extranodal organ (Fig. 4). The greatest median reductions were observed in lung, hepatobiliary, and pleural involvement, whereas bone, kidney, and CNS lesions demonstrated more variable responses.

Organ-level Cox regression revealed distinct patterns (Table 2). For PFS, pleural (HR 3.52, *p* = 0.003), hepatic (HR 2.01, *p* = 0.036), adrenal (HR 2.58, *p* = 0.037), and gastric involvement (HR 7.08, *p* = 0.012) was associated with increased risk of progression. For OS, pleural (HR 4.15, *p* = 0.0009), hepatic (HR 2.86, *p* = 0.0027), gastric (HR 6.44, *p* = 0.016), and muscular involvement (HR 1.93, *p* = 0.044) was associated with inferior survival. Hepatobiliary involvement was significantly associated with adverse outcomes at the organ system level (PFS HR 2.69, *p* = 0.0079; OS HR 3.69, *p* = 0.0007).

**Table 2:** Univariable cox regression

Organ	*n*	PFS events	OS events	PFS HR (95% CI)	PFS *p*-value	OS HR (95% CI)	OS *p*-value
Pleura	9	9	9	3.52 (1.60–6.97)	0.003*	4.15 (1.89–8.13)	< 0.001*
Lung	15	10	10	0.98 (0.47–1.81)	0.940	1.34 (0.64–2.54)	0.411
Pericardium	2	1	1	0.59 (0.03–2.02)	0.574	0.86 (0.05–3.91)	0.875
Hepatobiliary	14	13	13	2.69 (1.31–5.23)	0.008*	3.69 (1.78–7.33)	< 0.001*
Adrenal gland	7	7	6	2.58 (1.05–6.30)	0.037*	2.27 (0.87–4.90)	0.089
Ureter	1	1	1	0.74 (0.04–3.40)	0.762	1.19 (0.07–5.43)	0.866
Kidney	7	6	6	1.49 (0.58–3.17)	0.380	2.11 (0.81–4.54)	0.116
Stomach	3	3	3	7.08 (1.67–20.7)	0.012*	6.44 (1.52–18.6)	0.016*
Colon	2	1	1	0.53 (0.03–2.23)	0.481	0.66 (0.04–2.93)	0.657
Peritoneum	5	4	4	1.38 (0.42–3.32)	0.547	1.54 (0.46–3.75)	0.437
Muscle	19	14	14	1.40 (0.75–2.42)	0.280	1.93 (1.02–3.45)	0.044*
Bone	8	6	6	1.42 (0.55–3.01)	0.437	1.85 (0.71–3.96)	0.188
Fat	8	5	5	0.78 (0.27–1.76)	0.578	0.91 (0.32–2.07)	0.846
CNS	7	3	2	0.42 (0.10–1.13)	0.095	0.42 (0.07–1.30)	0.166
Orbit	1	1	1	3.80 (0.21–17.9)	0.279	9.31 (0.51–47.8)	0.106
Thyroid	2	2	1	1.88 (0.31–6.06)	0.426	0.54 (0.03–2.41)	0.498
LDH (per 100 U/L)	98	68	54	1.02 (1.00−1.04)	0.038*	1.02 (1.00–1.04)	0.018*
ECOG 1 versus 0	107	75	59	0.83 (0.53−1.31)	0.423	1.14 (0.68–1.91)	0.628
ECOG 2 versus 0	107	75	59	2.57 (1.37−4.81)	0.003*	1.97 (0.99–3.90)	0.052
ECOG 3 versus 0	107	75	59	2.06 (0.89−4.76)	0.090	3.00 (1.29–7.02)	0.011*
>= 2 extranodal lesions	109	76	60	1.64 (1.03−2.60)	0.036*	2.21 (1.33–3.67)	0.002*

In multivariable Cox regression including LDH, ECOG performance status, and ≥ 2 extranodal lesions (Supplementary Table S1), pleural involvement remained associated with both PFS and OS, and adrenal involvement with PFS, in this exploratory analysis; involvement of ≥ 2 extranodal sites showed an association with OS. Given the small number of organ-positive patients and the associated overfitting risk noted above, these multivariable estimates should be regarded as hypothesis-generating rather than confirmatory. Across these four multivariable models (6 parameters each: organ involvement, LDH, ECOG [3 dummy levels], ≥ 2 extranodal lesions), overall events-per-variable (EPV) was 12.7 for PFS (76 events/109 patients) and 10.0 for OS (60 events), meeting conventional thresholds (≥ 10); however, the small number of organ-positive patients (*n* = 7–19) combined with high within-subgroup event concentration (e.g., 9/9 pleura-involved patients had a PFS event) can independently destabilize the corresponding coefficient irrespective of overall model EPV. Table 2 reports patient numbers and event counts for each organ alongside the hazard ratios, together with univariable estimates for the clinical covariates used in the multivariable models.

Baseline SPD was associated with both PFS (HR per doubling 1.17 [95% CI 1.01–1.35], *p* = 0.041) and, more modestly, OS (HR 1.15 [0.98–1.35], *p* = 0.083) in univariable analysis of the extranodal group; this association was not observed in the nodal group (*p* = 0.32–0.35), likely reflecting reduced power upon stratification, and should be interpreted as hypothesis-generating given the exploratory nature of these stratified analyses.

CART products used in this cohort were axicabtagene ciloleucel (57.8%), tisagenlecleucel (30.3%), lisocabtagene maraleucel (11.0%), and one investigational construct (zamtocabtagene autoleucel, 0.9%); product use reflected sequential clinical availability over the study period (tisagenlecleucel predominating in earlier years, axicabtagene ciloleucel and lisocabtagene maraleucel more recently), precluding causal attribution of outcome differences to product selection per se.

### Survival analysis by extranodal sites and organ involvement

Median PFS in the overall cohort was 130 days (IQR, 90–260). Patients with nodal disease had longer PFS (306.5 days [IQR, 153–418]) than those with extranodal disease (90 days [IQR, 70–130]). Similarly, OS was longer in nodal disease (613 days [IQR, 333–860]) compared with extranodal disease (247 days [IQR, 169–384]).

**Fig. 5:**
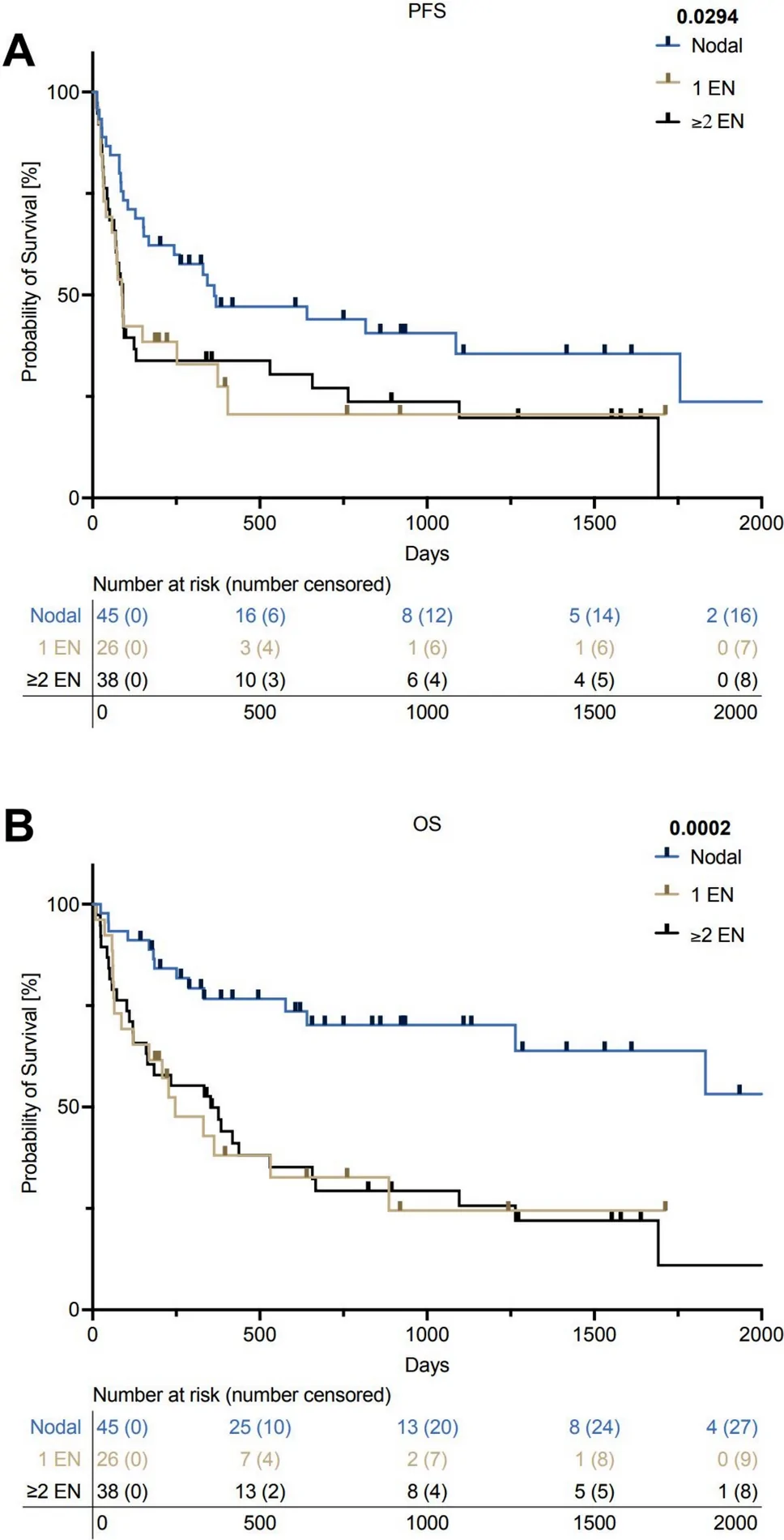
Kaplan–Meier survival curves according to extent of extranodal involvement. Curves show PFS (left) and OS (right) stratified by disease distribution: nodal only, 1 extranodal site, and ≥ 2 extranodal sites

**Fig. 6:**
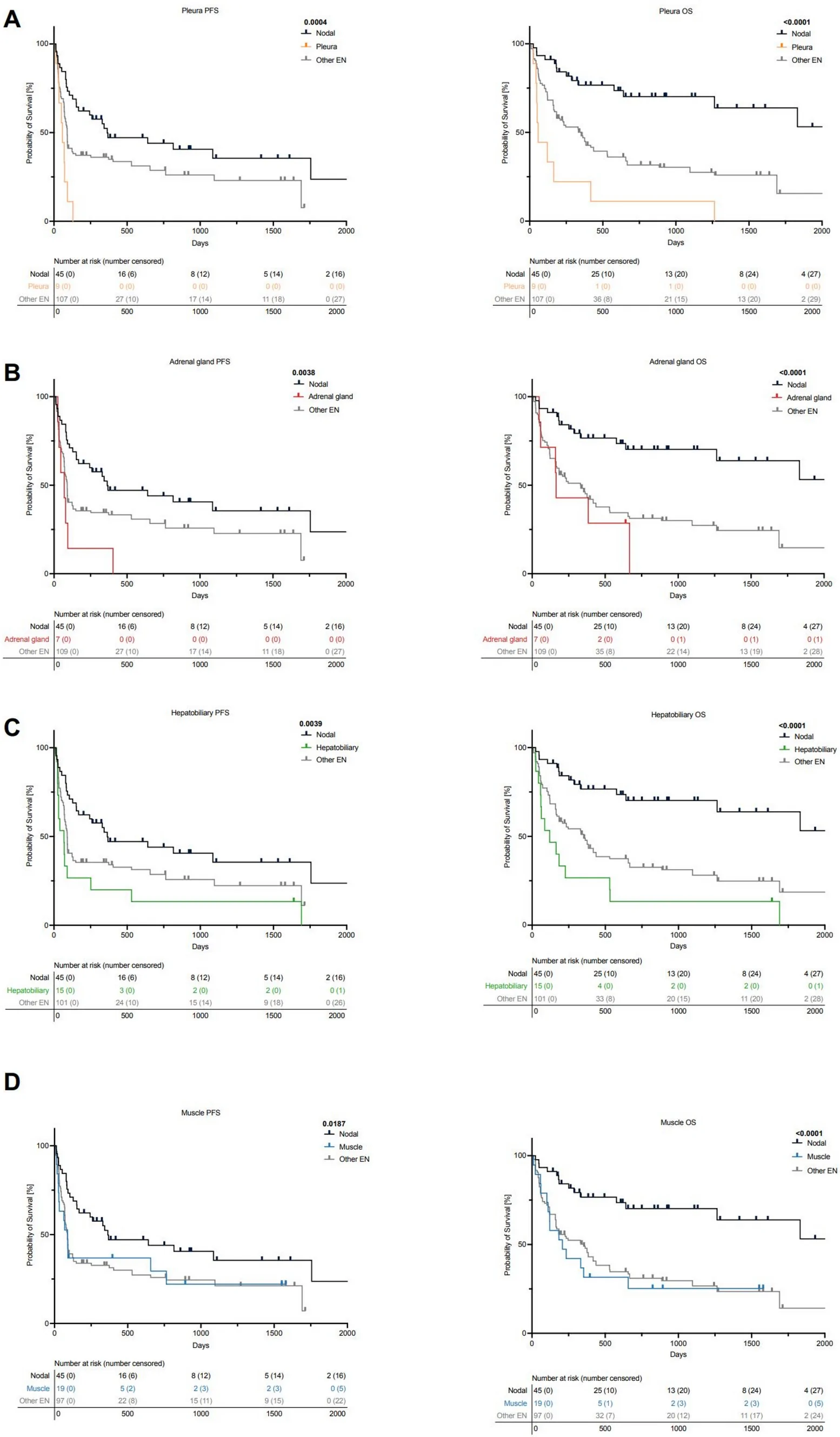
Site-specific survival according to extranodal involvement. Kaplan–Meier curves show PFS (left column) and OS (right column) stratified by specific extranodal disease sites (pleura, adrenal gland, hepatobiliary system, muscle)

Both PFS and OS were reduced in patients with ≥ 2 extranodal sites compared with nodal disease (*P* = 0.029 for PFS; *P* = 0.0002 for OS) (Fig. 5). In this exploratory analysis, Kaplan–Meier curves for candidate high-risk compartments suggested a stepwise pattern, with nodal-only disease showing the most favorable outcomes and comparatively poorer outcomes observed in patients with pleural, adrenal, or hepatobiliary involvement (Fig. 6), though patient numbers at risk for individual sites were small (*n* = 7–19; see Table 2). Pleural involvement showed early separation of survival curves from nodal disease, and hepatobiliary involvement showed a comparable pattern, whereas muscle involvement showed no clear survival separation from other extranodal organs.

## Discussion

In this study, we investigated spatial patterns of disease involvement and compartment-specific response dynamics in patients with LBCL using longitudinal imaging. Our findings demonstrate three principal observations: (1) extranodal disease is common and associated with inferior survival compared with purely nodal disease; (2) tumor burden reduction occurs across both nodal and extranodal compartments but does not fully explain survival differences; and (3) specific extranodal organs, particularly hepatobiliary, pleural, and adrenal involvement, emerged as candidate high-risk spatial phenotypes in this exploratory analysis, with disproportionate impact on survival, warranting confirmation in larger, adequately powered cohorts.

The principal novelty of this study lies in the integration of organ-specific tumor burden quantification with survival analysis across individual anatomical compartments. While most imaging studies in lymphoma have focused on global tumor metrics, such as total tumor volume or metabolic tumor burden, our analysis demonstrates that the spatial distribution of disease across organ systems provides information beyond overall tumor reduction. Importantly, these observations were derived from routine CT-based response assessment rather than advanced imaging techniques, suggesting that spatial disease phenotyping may represent a broadly accessible imaging biomarker in clinical practice.

The prognostic importance of extranodal disease has long been recognized in lymphoma staging systems including the Ann Arbor classification and IPI. However, these systems typically incorporate only the presence or number of extranodal sites, rather than the specific organs involved [16]. More recently, studies have begun to explore the importance of anatomical disease patterns in aggressive lymphoma. Iacoboni et al. [17] analyzed extranodal disease patterns in a large CART cohort and demonstrated that coexistence of nodal and extranodal disease and increasing numbers of extranodal sites were associated with inferior clinical outcomes. Liver and adrenal involvement was identified as particularly adverse locations. Our findings closely parallel these observations. Further evidence for organ-specific heterogeneity has been reported by Luna et al. [5], who demonstrated significant variability in site-specific response rates across anatomical compartments, with particularly unfavorable outcomes in adrenal/genitourinary and pleural manifestations.

One of the most notable findings of our study is that the magnitude of morphologic tumor reduction was comparable between nodal and extranodal compartments. Median DoR exceeded 75% in both compartments by the second follow-up examination, indicating effective cytoreduction at the global disease level. However, survival differences persisted depending on the anatomical site of disease involvement, suggesting that size-based response metrics alone may not fully capture biologic disease heterogeneity. In recent years, quantitative PET imaging biomarkers, including metabolic tumor volume (MTV), have been increasingly investigated for risk stratification in LBCL [18–21]. Beyond global tumor burden metrics, imaging-based analyses of spatial heterogeneity have also shown promising results [22, 23].

The consistent identification of hepatobiliary, adrenal, and thoracic serosal compartments as high-risk sites across multiple studies raises important biological considerations. The liver is known to maintain an immunologically tolerant microenvironment that may facilitate tumor immune escape [24]. The hepatic immune environment has been shown to promote tumor tolerance through regulatory immune cell populations and immunosuppressive cytokine signaling [25]. Serosal surfaces such as the pleura provide specialized stromal and vascular microenvironments that may facilitate tumor cell dissemination and persistence [26].

This study has several limitations. First, the retrospective single-center design may introduce selection bias and limits generalizability. The extranodal and nodal groups also differ systematically at baseline (higher LDH, more advanced Ann Arbor stage, higher IPI, and greater tumor burden in the extranodal group; Table 1), and these factors may confound the site-specific survival associations reported here. Second, the sample size for certain organ-specific subgroups was relatively small, which may have reduced statistical power. Sixteen organ sites were evaluated across two endpoints (32 comparisons) without correction for multiple testing, and organ-level events-per-variable, while meeting conventional thresholds overall, do not fully capture the instability introduced by small, high-event-concentration subgroups; these findings should be regarded as exploratory and hypothesis-generating rather than confirmatory. Third, tumor burden was assessed using morphologic CT-based measurements rather than metabolic imaging metrics such as PET-derived MTV. Differential lesion detection sensitivity between CT and PET/CT cannot be fully excluded as a source of bias in a mixed-modality cohort. Fourth, treatment regimens were not uniform across the cohort. Additionally, peri-CART treatment strategies, including bridging radiotherapy, were not incorporated into the present analysis and may represent potential unaccounted confounders of extranodal response and survival outcomes. Extranodal patients were also more likely to be excluded from second follow-up imaging than nodal patients, predominantly due to early death; the reported comparability of depth of response between groups at this timepoint should be interpreted in light of this survivor-enriched subset. Finally, the observational design does not allow mechanistic conclusions regarding the biological drivers of organ-specific prognostic differences.

In conclusion, extranodal disease in LBCL appears to show substantial spatial heterogeneity in both treatment response and clinical outcome. While morphologic tumor reduction occurs across nodal and extranodal compartments, survival outcomes may be influenced by the specific organs involved. Consistent with recent imaging and CART cohort studies, hepatobiliary, pleural, and adrenal involvement appears to represent candidate high-risk spatial disease phenotypes that warrant confirmation in larger, adequately powered, multicenter cohorts. These findings suggest a potential role for CT-based spatial disease phenotyping as an accessible imaging biomarker that may complement established metabolic imaging approaches in LBCL.

## Supplementary Information

Below is the link to the electronic supplementary material.

Supplementary file1 (DOCX 117 KB)

## Data Availability

No datasets were generated or analysed during the current study.
