# Supplementary material for: Organ-specific depth of response to CD19-directed CAR T-cell therapy in B-cell lymphomas
Source: Cancer Immunol Immunother. 2026 Oct 25;75(11):254. doi: 10.1007/s00262-026-04605-7 (PMC13710507; doi:10.1007/s00262-026-04605-7)
Supplement: Supplementary file 1 [file 262_2026_4605_MOESM1_ESM.docx]

**SUPPLEMENTAL MATERIALS**

**Supplementary Figure 1: Patient inclusion flowchart.** Flow diagram showing cohort selection and availability of imaging follow-up. From 160 patients with non-Hodgkin lymphoma (NHL), 122 patients with large B-cell lymphoma (LBCL) were identified. Baseline imaging was available for 109 patients. Imaging follow-up was available in 102 patients at first follow-up and 84 patients at second follow-up. Patients were excluded during follow-up due to death or unavailable imaging data.

**Supplementary Table 1: Multivariable Cox Regression.**

**Pleural involvement model**

| **Variable** | **PFS HR** | **PFS 95% CI** | **PFS p** | **OS HR** | **OS 95% CI** | **OS p** |
| --- | --- | --- | --- | --- | --- | --- |
| Pleural involvement | 3.10 | 1.26–6.91 | 0.016 | 2.83 | 1.15–6.36 | 0.025 |
| LDH | 1.00 | 0.999–1.00 | 0.521 | 0.9997 | 0.9985–1.00 | 0.111 |
| ECOG 1 vs. 0 | 0.83 | 0.44–1.63 | 0.589 | 0.85 | 0.39–1.90 | 0.675 |
| ECOG 2 vs. 0 | 1.17 | 0.46–2.85 | 0.733 | 1.20 | 0.41–3.33 | 0.737 |
| ECOG 3 vs. 0 | 1.97 | 0.69–4.99 | 0.193 | 1.78 | 0.53–5.22 | 0.330 |
| ≥2 extranodal lesions | 1.29 | 0.71–2.32 | 0.391 | 2.03 | 1.04–3.99 | 0.037 |

**Adrenal involvement model**

| **Variable** | **PFS HR** | **PFS 95% CI** | **PFS p** | **OS HR** | **OS 95% CI** | **OS p** |
| --- | --- | --- | --- | --- | --- | --- |
| Adrenal involvement | 2.85 | 1.06–6.49 | 0.039 | 2.89 | 0.94–7.93 | 0.062 |
| LDH | 1.00 | 0.9997–1.00 | 0.712 | 0.9996 | 0.9980–1.00 | 0.056 |
| ECOG 1 vs. 0 | 1.00 | 0.52–1.99 | 0.999 | 1.01 | 0.47–2.29 | 0.983 |
| ECOG 2 vs. 0 | 1.23 | 0.47–3.02 | 0.666 | 1.22 | 0.42–3.46 | 0.708 |
| ECOG 3 vs. 0 | 2.11 | 0.72–5.47 | 0.162 | 1.87 | 0.55–5.65 | 0.300 |
| ≥2 extranodal lesions | 1.49 | 0.85–2.61 | 0.167 | 2.40 | 1.28–4.61 | 0.0065 |

**Hepatobiliary involvement model**

| **Variable** | **PFS HR** | **PFS 95% CI** | **PFS p** | **OS HR** | **OS 95% CI** | **OS p** |
| --- | --- | --- | --- | --- | --- | --- |
| Hepatobiliary involvement | 1.62 | 0.74–3.26 | 0.213 | 1.86 | 0.85–3.80 | 0.119 |
| LDH | 1.00 | 0.9998–1.00 | 0.600 | 0.9997 | 0.9986–1.00 | 0.105 |
| ECOG 1 vs. 0 | 0.90 | 0.48–1.74 | 0.744 | 0.92 | 0.44–2.03 | 0.824 |
| ECOG 2 vs. 0 | 1.03 | 0.39–2.57 | 0.948 | 0.98 | 0.33–2.77 | 0.969 |
| ECOG 3 vs. 0 | 1.79 | 0.62–4.53 | 0.263 | 1.49 | 0.44–4.40 | 0.495 |
| ≥2 extranodal lesions | 1.39 | 0.77–2.48 | 0.266 | 2.17 | 1.13–4.20 | 0.020 |

**Muscle involvement model**

| **Variable** | **PFS HR** | **PFS 95% CI** | **PFS p** | **OS HR** | **OS 95% CI** | **OS p** |
| --- | --- | --- | --- | --- | --- | --- |
| Muscle involvement | 1.18 | 0.57–2.37 | 0.643 | 1.17 | 0.56–2.36 | 0.673 |
| LDH | 1.00 | 0.9998–1.00 | 0.600 | 0.9997 | 0.9987–1.00 | 0.100 |
| ECOG 1 vs. 0 | 0.90 | 0.48–1.74 | 0.742 | 0.91 | 0.43–2.02 | 0.813 |
| ECOG 2 vs. 0 | 1.17 | 0.46–2.83 | 0.738 | 1.13 | 0.39–3.12 | 0.822 |
| ECOG 3 vs. 0 | 1.87 | 0.65–5.47 | 0.232 | 1.61 | 0.48–4.73 | 0.416 |
| ≥2 extranodal lesions | 1.43 | 0.75–2.67 | 0.276 | 2.30 | 1.14–4.67 | 0.021 |

**Supplementary Table 2: Events-per-variable (EPV) summary**

This summary confirms the number of events relative to covariates in the multivariable models above. All four models share an identical structure: organ involvement, LDH, ECOG (3 dummy levels vs. reference 0), and ≥2 extranodal lesions (6 parameters total).

| **Endpoint** | **N** | **Total events** | **Parameters** | **EPV** |
| --- | --- | --- | --- | --- |
| PFS | 109 | 76 | 6 | 12.7 |
| OS | 109 | 60 | 6 | 10.0 |

*Note: overall EPV meets conventional thresholds (≥10). However, given the small number of organ-positive patients (n=7–19 across models) and high within-subgroup event concentration, individual organ coefficients may remain unstable irrespective of overall model EPV; see main manuscript Discussion for further caveats.*
